# Global burden of chronic kidney disease due to diabetes mellitus, 1990-2021, and projections to 2050

**DOI:** 10.3389/fendo.2025.1513008

**Published:** 2025-02-21

**Authors:** Xiao Ma, Rong Liu, Xiang Xi, Hui Zhuo, Yiwei Gu

**Affiliations:** Department of Urology, The Third People's Hospital of Chengdu, Chengdu, Sichuan, China

**Keywords:** chronic kidney disease, global disease burden, years lived with disability, health inequality, diabetes mellitus, diabetic nephropathy

## Abstract

**Introduction:**

Chronic kidney disease (CKD) is a serious complication of diabetes, and the global burden of the disease is gradually increasing.

**Methods:**

This study systematically analyzed the trends and future projections of the worldwide burden of chronic kidney disease caused by type 1 and type 2 diabetes mellitus based on the Global Burden of Disease Study (GBD) using data from 1990 to 2021. Number of deaths, Age-standardized mortality rates, disability-adjusted life years (DALYs), and age-standardized DALYs rate were used to estimate the disease burden. The study used Estimated Annual Percentage Changes (EAPCs) to calculate trends in the burden of each disease subtype and different regions and assessed the impact of various age groups and metabolic factors on chronic kidney disease due to diabetes. The ARIMA model was further used to predict the burden of Diabetic nephropathy from 2022 to 2050.

**Results:**

The results of the study showed that the burden of Diabetic nephropathy and its EAPCs varied significantly in distribution across different Sociodemographic Index subgroups of countries as well as among 204 countries and regions worldwide. In addition, the influence of different age groups and metabolic factors on the burden of Diabetic nephropathy also showed significant variability. The effects of metabolic factors on the number of deaths and mortality were positively correlated with age. Different metabolic factors have different effects on the mortality of CKD due to type 1 diabetes and CKD due to type 2 diabetes. The ARIMA model predicts that the global burden of Diabetic nephropathy will continue to increase in the absence of interventions.

**Conclusions:**

To effectively manage CKD caused by diabetes, more accurate and cost-effective diagnostic tools and interventions are needed in the future, especially in low - and middle-income countries with poor healthcare resources.

## 1 Introduction

Chronic kidney disease (CKD) is defined as abnormalities of kidney structure or function, present for greater than 3 months with specific implications for health, and has become one of the serious public health problems (1). The diagnostic criteria for CKD are: 1. Signs of renal injury (presence of any one of the following for more than 3 months): (1) albuminuria (urinary albumin/creatinine ratio UACR ≥ 30 mg/g, or urinary albumin excretion rate UAER ≥ 30 mg/24h); (2) abnormal urinary sediment;3renal tubule-related lesions; (4) histologic abnormalities;(5) structural seen on imaging abnormalities; and (6) history of renal transplantation.2. Decreased renal function: estimated glomerular filtration rate (eGFR)<60 ml-min-¹-(1.73 m²)-¹ for more than 3 months (2). Diabetic nephropathy (DN) is a common long-term complication of Type 1 and Type 2 diabetes (3). DN is a CKD caused by diabetes (4). DN is an important type of CKD that increases mortality in diabetics (5). Studies have found that nearly half of patients with type 2 diabetes and one-third of patients with type 1 diabetes can progress to CKD (6). Diabetes is a known risk factor for CKD, and the prevalence of diabetes in patients with CKD ranges from 31 to 40% (7). It is less likely to cause death directly, and serious complications such as diabetic kidney disease are the main culprits of death and disability in patients with diabetes (8). Between 2000 and 2019, nearly 800,000 patients in the United States underwent dialysis or transplantation for kidney failure, with diabetes as the leading cause (9). It is estimated that more than one-fifth of healthcare expenditures in the United States are primarily for type 2 diabetes, with the majority focused on the management of complications in patients with type 2 diabetes. The most prominent complication of type 2 diabetes is CKD (10). The 2017 Global Burden of Disease study showed that the global prevalence of CKD is 9.1% with approximately 70 billion cases. Since 1990, the prevalence of CKD has increased by 29.3%. However, the burden of CKD is not uniform across regions of the globe, and countries with different incomes are affected differently. Patients from low-to-middle-income countries are often the least able to deal with the burden of DN and the healthcare costs of the disease. Patients in low - to middle-income countries cannot cope with the burden of diabetic kidney disease, and most healthcare facilities in these countries cannot adequately cope with the special needs of precision renal care (11). Independent risk factors for DN are currently considered to include genetics, hyperglycemia, hypertension, dyslipidemia, and proteinuria (12). Although there are no complete statistics on the incidence of diabetes complicated with CKD, the relevant data play an important role in finding the risk population, evaluating the effectiveness of management programs, and assessing the harm of DN (13).

This paper focuses on the trends and future predictions of deaths, DALYs, etc. in CKD due to diabetes mellitus type 2 and CKD due to diabetes mellitus type 1, and the relationship with the metabolic factors (Kidney dysfunction, High fasting plasma glucose, High body-mass index, etc.) These data can inform the development of cost-effective policies and tailored interventions for diabetic CKD, especially in populations at high risk for diabetic kidney disease.

## 2 Method

### 2.1 Data sources

Mortality, number of deaths, and disability-adjusted life year (DALYs) rates for chronic kidney disease caused by T1DM and T2DM were extracted in the GBD Database 2021 study. We obtained disease data from the GBD database for 1990-2021(http://ghdx.healthdata.org/gbd-results-tool). The GBD 2021 study covers the health burden of 371 diseases and injuries in 204 countries and territories, covering age, gender, country, and region. Global and country-level data are available for each year from 1990 to 2021 (14). The GBD database collects health survey data, hospital records, clinical research data, disease registry system data, death records, national health statistics reports, data released by international organizations, academic research papers, and many other data types from all countries (15). The disease burden of CKD was expressed in terms of mortality and DALYs. Global data on chronic kidney disease caused by T1DM and T2DM were collected: number of deaths, age-standardized mortality (ASMR), DALYs, and age-standardized DALYs rates (16). The incidence of chronic kidney disease refers to the incidence per 100,000 people. We use age-standardized rates to account for the rapid growth of the population and the diverse age composition. Among them, DALY combined with years of life lost (YLL) and years lost due to disability (YLD) was used to measure the burden of chronic kidney disease caused by T1DM and T2DM. The GBD database uses Standard Life Table, DisMod-MR 2.1 model and other methods to integrate data from multiple sources and adjust morbidity and mortality rates for consistency, thus improving the accuracy and comparability of the data (17).

### 2.2 The sociodemographic index

SDI is a composite indicator that measures the level of socio-economic development of a country or region. It is calculated based on three core factors: per capita income, education level, and total fertility rate. The SDI ranges from 0 to 1, with higher values indicating higher levels of socio-economic development of a region. In this paper, SDI was divided into five categories, among others, to better analyze the burden of DN in countries and regions with different levels of socioeconomic development. The countries and territories covered by the GBD are categorized into five SDI categories based on the following ranges: low SDI (0 to 0.4547), medium-low SDI (0.4547 to 0.6076), medium SDI (0.6076 to 0.7905), medium-high SDI (0.7905 to 0.8051), and high SDI (0.8051 to 1) (18).

### 2.3 ARIMA model

The ARIMA model is a statistical model commonly used in time series analysis and forecasting. It captures the characteristics of the data in a time series by combining autoregressive (AR), difference (I), and moving average (MA) components. The form of an ARIMA model is usually denoted as ARIMA (p, d, q), where p denotes the order of the autoregressive term, d denotes the number of differencing required to smooth the time series, and q denotes the order of the moving average term. The AR part represents the linear relationship between the current data point and its previous p data points, the MA part reflects the linear combination of the current data point and the previous q residuals, and the I part removes the trend by performing d times of differencing on the data by performing a d-difference to remove the trend. The basic mathematical formula of the ARIMA model can be expressed as: $y_{t}=c+\phi_{1}y_{t-1}+\phi_{2}y_{t-2}+\cdots +\phi_{p}y_{t-p}+\theta_{1}\epsilon_{t-1}+\theta_{2}\epsilon_{t-2}+\cdots +\theta_{q}\epsilon_{t-q}+\epsilon_{t},$ . By adjusting the parameters “p, d, q” of the ARIMA model, different types of time series can be fitted, which can be used to predict future data points (19).

### 2.4 ARIMAX model

ARIMAX is an extension of the ARIMA model to improve the dynamics and accuracy of forecasting by introducing external variables (i.e., explanatory variables) into the model (20). The basic form of the ARIMAX model is:


$$Y_{t}\;=\;\alpha \;+\;\sum_{i=1}^{p}\;\phi_{i}Y_{t-i}\;+\;\sum_{j=1}^{q}\;\theta_{j}\epsilon_{t-j}\;+\;\sum_{k=1}^{r}\;\beta_{k}X_{t-k}\;+\;\epsilon_{t}$$

$Y_{t}$ is the target variable, $X_{t}$ is the external variable, α is the constant term, $\phi_{i}$ and $\theta_{j}$ are the coefficients of the autoregressive (AR) and moving average (MA) terms, respectively, and $\beta_{k}$ is the coefficient of the external variable.

### 2.5 Estimated average annual percentage changes

EAPCs a statistics commonly used to describe the trend over time of a health indicator such as morbidity, mortality, or disability-adjusted life year rates. It is used to measure the rate of percentage change of an indicator over a while and is particularly useful for observing trends over time. The calculation of EAPCs is based on linear regression on time-series data, using the formula:


$$\text{ln}(Y)=\beta_{0}+\beta_{1}\cdot t,$$

Where Y denotes some health indicator, and  t is the year, and ${\text{ β}}_{1}$ is the regression coefficient, reflecting the trend of change from year to year, and ${\text{β}}_{0}$ is the intercept term in the linear regression equation. EAPC can be calculated by using the equation


$$EAPC=\left(e^{{\text{β}}_{1}}-1\right)\times 100\%$$

Is computed, where  e is the base of the natural logarithm, and ${\text{ β}}_{1}$ is the estimated annual rate of change (EAPC). A positive EAPC indicates an upward trend in the indicator over the observation period, while a negative EAPC indicates a downward trend, and the size of the absolute value of the EAPC reflects the speed of change.

## 3 Results

### 3.1 Global rates of age-standardized DALYs for DN, 1990 and 2021

**Table 1** shows the age-standardized rates of DALYs for DN by sex, disease subtype, SDI, and region in 1990 and 2021 (per 100,000 population). In terms of global trends, the global rate of DALYs for DN is generally increasing from 1990 to 2021, with a more significant increase in type 2 DN, but a slight decrease in the rate of DALYs in women with type 1 DN. The DALYs rate for type 1 DN was 51.439/100,000 and 42.693/100,000 in 1990 and 53.209/100,000 and 27.273/100,000 in 2021 for men and women, respectively. The rates of DALYs for type 2 DN in 1990 were 121.653/100,000 and 93.543/100,000 for males and females, respectively, rising to 1,518,130/100,000 and 1,136,710/100,000 by 2021, respectively. Overall, the burden of disease for DN in 2021 is significantly higher than in 1990.

**Table 1:** Global rates of age-standardized DALYs for DN by sex, disease subtype, SDI, and region, 1990 and 2021 (per 100,000 population).

1990						
	Chronic kidney disease due to diabetes mellitus type 1			Chronic kidney disease due to diabetes mellitus type 2		
	Male	Female	Both	Male	Female	Both
**Global**	51.439	42.693	47.048	121.653	93.543	105.710
SDI quintile						
High SDI	19.862	13.561	16.636	73.147	55.278	62.473
High-middle SDI	44.043	35.708	39.818	92.769	69.303	78.208
Middle SDI	75.074	68.553	71.883	167.755	149.868	157.871
Low-middle SDI	52.538	41.784	47.322	143.054	106.541	125.032
Low SDI	64.655	49.532	57.256	192.158	140.093	166.426
Eastern Europe &Central Asia						
Central Asia	10.407	7.531	8.897	47.247	41.853	44.137
Central Europe	14.432	7.285	10.71	44.161	27.169	34.129
Eastern Europe	19.188	18.111	18.581	21.647	21.33	21.232
High-income						
Australasia	4.227	3.591	3.9	19.453	17.839	18.291
High-income Asia Pacific	27.528	17.38	22.279	111.401	79.508	92.691
High-income North America	14.986	9.564	12.203	71.727	59.944	64.851
Southern Latin America	36.765	21.178	28.549	141.393	83.676	108.595
Western Europe	10.003	5.548	7.675	48.35	32.646	38.703
Latin America & Caribbean						
Andean Latin America	74.844	52.43	63.49	232.825	187.775	209.719
Caribbean	90.443	47.224	68.419	217.467	132.683	173.443
Central Latin America	42.328	38.777	40.51	152.952	144.181	148.562
Tropical Latin America	63.624	38.224	50.555	176.658	115.327	143.569
South Asia & North Africa and Middle East						
North Africa and Middle East	20.03	19.88	19.958	140.712	137.471	139.127
South Asia	46.508	28.552	38.034	132.38	85.579	110.085
Southeast Asia, East Asia and Oceania						
East Asia	82.238	80.096	81.197	164.64	158.137	158.732
Oceania	147.299	115.272	131.899	285.641	215.801	250.325
Southeast Asia	108.57	103.94	106.132	210.436	177.773	192.494
Sub-Saharan Africa						
Central Sub-Saharan Africa	76.901	44.722	59.805	252.599	137.366	191.568
Eastern Sub-Saharan Africa	106.176	85.332	95.752	307.035	225.337	266.266
Southern Sub-Saharan Africa	34.873	19.001	26.399	103.675	62.644	79.524
Western Sub-Saharan Africa	37.973	26.866	32.679	133.925	98.212	115.498
2021						
	Chronic kidney disease due to diabetes mellitus type 1			Chronic kidney disease due to diabetes mellitus type 2		
	Male	Female	Both	Male	Female	Both
**Global**	53.209	37.273	45.204	151.813	113.671	131.082
SDI quintile						
High SDI	23.709	15.955	19.824	118.943	88.995	102.648
High-middle SDI	36.356	26.039	31.25	97.477	75.576	84.706
Middle SDI	72.649	51.187	61.887	191.602	146.41	167.063
Low-middle SDI	62.672	42.855	52.635	181.971	131.04	155.334
Low SDI	58.553	40.563	49.475	184.514	138.65	160.879
Eastern Europe & Central Asia						
Central Asia	17.42	10.69	13.915	79.696	58.4	67.497
Central Europe	11.435	5.828	8.56	42.337	26.951	33.452
Eastern Europe	20.375	17.214	18.725	25.232	27.433	26.691
High-income						
Australasia	6.185	5.086	5.62	26.299	21.299	23.595
High-income Asia Pacific	20.287	10.836	15.551	96.597	57.199	75.186
High-income North America	29.401	19.466	24.294	197.727	153.979	174.043
Southern Latin America	28.236	17.333	22.507	124.877	80.61	99.579
Western Europe	10.04	5.887	7.922	48.729	34.545	40.857
Latin America & Caribbean						
Andean Latin America	87.289	61.826	74.292	309.603	264.563	286.11
Caribbean	123.783	68.013	95.391	292.724	198.927	242.944
Central Latin America	92.722	64.183	77.718	265.33	204.651	232.617
Tropical Latin America	57.562	31.56	43.945	198.344	122.242	155.856
South Asia & North Africa and Middle East						
North Africa and Middle East	22.807	22.872	22.831	167.045	173.251	170.156
South Asia	55.548	33.334	44.485	163.993	106.406	134.388
Southeast Asia, East Asia and Oceania						
East Asia	57.702	40.008	49.093	13.991	112.915	125.582
Oceania	176.392	139.747	158.332	342.634	276.981	309.8
Southeast Asia	128.231	105.046	116.452	264.825	214.453	237.679
Sub-Saharan Africa						
Central Sub-Saharan Africa	75.47	44.758	59.507	257.694	148.944	196.095
Eastern Sub-Saharan Africa	82.354	56.068	68.875	267.917	197.901	230.386
Southern Sub-Saharan Africa	44.989	24.812	34.177	137.852	89.456	108.87
Western Sub-Saharan Africa	43.381	25.948	34.218	145.64	105.214	124.262

Considering the different SDI countries, the DALYs rate of DN was lowest in High SDI countries in 1990, with type 1 DN and type 2 DN of 16.636/100,000 and 62.473/100,000, respectively, and in 2021 it rose to 19.824/100,000 and 102.648/100,000, the rate of DALYs for type 2 DN is rapidly increasing and has surpassed that of High-middle SDI countries, reflecting the fact that High SDI countries are under pressure from the increased burden of DN. DALYs rates for DN have remained consistently high in middle SDI countries, with higher overall DALYs rates in 1990 and 2020 in middle SDI countries than in other SDI countries.

Of the 21 different regions, East and Central Asia and the high-income regions had lower DALYs rates, with Australasia consistently having lower DALYs rates than all other regions except for men with type 2 DN in 2021. The rate of DALYs for type 2 DN in high-income North America increased rapidly from 1990 to 2021, from 64.851 per 100,000 to 174.043 per 100,000 people. To 2021, increasing rapidly from 64.851 per 100,000 to 174.043 per 100,000 people. Latin America and Caribbean region, Southeast Asia, East Asia and Oceania region, and the central and eastern regions of Sub-Saharan Africa are all regions with high prevalence of DN, and Oceania in particular has consistently high DALYs rates in 1990 and 2021. high rates of DALYs, and this consistently high level of burden suggests that the region faces serious challenges in disease management. Compared with 1990, the rates of DALYs for both types of DN in 2021 decreased significantly in Oceania from 81.197/100,000 and 158.732/100,000 to 49.093/100,000 and 125.582/100,000, respectively, reflecting better disease control.

### 3.2 Age-standardized mortality rates for DN, 1990 and 2021

Age-standardized mortality rates (per 100,000) for DN by sex, subtype, SDI, and region in 1990 and 2021 are shown in **Table 2**. There was little overall change in DN mortality rates in 1990 and 2021. Global mortality rates for type 2 DN, as well as for high-SDI countries and medium- and low-SDI countries showed a slight increase in mortality. There was also a consistent distribution of DN mortality and DALYs rates across countries and regions. Regions with low mortality rates include Eastern Europe & Central Asia, Australasia and Western Europe, while regions with high mortality rates include Latin America & Caribbean, Southeast Asia, and East Asia, and High mortality rates are found in Latin America & Caribbean, Southeast Asia, East Asia and Oceania, and the central and eastern parts of Sub-Saharan Africa. The region with the highest increase in mortality is High-income North America, where the mortality rate for type 2 DN increased from 2.245/100,000 people in 1990 to 8.082/100,000 people in 2021.

**Table 2:** Age-standardized mortality rates for DN by sex, disease subtype, SDI, and region, globally, 1990 and 2021 (per 100,000 population).

1990						
	Chronic kidney disease due to diabetes mellitus type 1			Chronic kidney disease due to diabetes mellitus type 2		
	Male	Female	Both	Male	Female	Both
**Global**	1.191	0.972	1.08	4.979	3.598	4.154
SDI quintile						
High SDI	0.402	0.27	0.333	2.845	2.091	2.364
High-middle SDI	0.982	0.797	0.886	3.89	2.54	3.005
Middle SDI	1.735	1.586	1.661	7.356	6.333	6.766
Low-middle SDI	1.332	1.013	1.177	5.625	4.176	4.901
Low SDI	1.718	1.284	1.506	8.43	5.906	7.165
Eastern Europe & Central Asia						
Central Asia	0.169	0.125	0.146	0.659	0.605	0.634
Central Europe	0.346	0.173	0.254	1.521	0.829	1.088
Eastern Europe	0.464	0.448	0.454	0.393	0.299	0.316
High-income						
Australasia	0.074	0.061	0.068	0.599	0.463	0.505
High-income Asia Pacific	0.581	0.356	0.462	5.236	3.89	4.408
High-income North America	0.264	0.167	0.213	2.5	2.094	2.245
Southern Latin America	0.981	0.528	0.739	6.803	4.035	5.182
Western Europe	0.204	0.109	0.153	1.804	1.167	1.383
Latin America & Caribbean						
Andean Latin America	2.029	1.442	1.731	11.372	9.5	10.406
Caribbean	2.217	1.202	1.699	9.717	6.377	7.958
Central Latin America	1.11	1.026	1.067	6.397	6.984	6.717
Tropical Latin America	1.583	0.946	1.254	7.646	5.044	6.201
South Asia & North Africa and Middle East						
North Africa and Middle East	0.488	0.476	0.482	6.347	6.353	6.35
South Asia	1.224	0.752	1.001	4.96	3.165	4.098
Southeast Asia, East Asia and Oceania						
East Asia	1.81	1.804	1.806	7.856	6.653	6.988
Oceania	3.437	2.643	3.055	12.788	8.854	10.709
Southeast Asia	2.51	2.306	2.403	8.815	7.16	7.876
Sub-Saharan Africa						
Central Sub-Saharan Africa	2.141	1.169	1.624	11.488	6.018	8.598
Eastern Sub-Saharan Africa	2.877	2.25	2.565	14.747	10.55	12.626
Southern Sub-Saharan Africa	0.848	0.434	0.624	4.325	2.018	2.915
Western Sub-Saharan Africa	0.995	0.692	0.85	6.065	3.925	4.92
2021						
	Chronic kidney disease due to diabetes mellitus type 1			Chronic kidney disease due to diabetes mellitus type 2		
	Male	Female	Both	Male	Female	Both
**Global**	1.278	0.895	1.084	6.77	4.926	5.723
SDI quintile						
High SDI	0.518	0.33	0.422	5.387	4.028	4.618
High-middle SDI	0.816	0.597	0.706	4.461	3.152	3.653
Middle SDI	1.745	1.239	1.49	8.859	6.484	7.509
Low-middle SDI	1.617	1.095	1.351	7.556	5.451	6.433
Low SDI	1.541	1.076	1.306	8.497	6.329	7.363
Eastern Europe & Central Asia						
Central Asia	0.326	0.193	0.256	2.233	1.436	1.76
Central Europe	0.259	0.127	0.19	1.521	0.906	1.147
Eastern Europe	0.546	0.49	0.518	0.679	0.803	0.767
High-income						
Australasia	0.084	0.064	0.074	0.969	0.702	0.822
High-income Asia Pacific	0.4	0.178	0.287	4.566	2.859	3.603
High-income North America	0.71	0.444	0.572	9.212	7.19	8.082
Southern Latin America	0.743	0.434	0.578	6.339	4.137	5.035
Western Europe	0.157	0.09	0.122	2.123	1.582	1.807
Latin America & Caribbean						
Andean Latin America	2.467	1.824	2.138	15.885	13.937	14.858
Caribbean	3.096	1.802	2.434	13.393	9.685	11.38
Central Latin America	2.473	1.762	2.098	11.203	9.124	10.069
Tropical Latin America	1.496	0.82	1.139	9.27	5.712	7.219
South Asia & North Africa and Middle East						
North Africa and Middle East	0.566	0.565	0.565	7.839	8.529	8.187
South Asia	1.463	0.894	1.178	6.542	4.119	5.265
Southeast Asia, East Asia and Oceania						
East Asia	1.266	0.889	1.081	7.316	4.938	5.826
Oceania	4.13	3.274	3.711	15.581	11.794	13.628
Southeast Asia	3.027	2.425	2.718	11.474	9.43	10.353
Sub-Saharan Africa						
Central Sub-Saharan Africa	2.096	1.186	1.615	11.939	6.909	9.006
Eastern Sub-Saharan Africa	2.215	1.531	1.863	14.018	10.358	12.013
Southern Sub-Saharan Africa	1.137	0.612	0.851	5.855	3.435	4.351
Western Sub-Saharan Africa	1.121	0.669	0.883	6.581	4.507	5.481

### 3.3 Trends in the global burden of DN between 1990 and 2050

**Figure 1:**
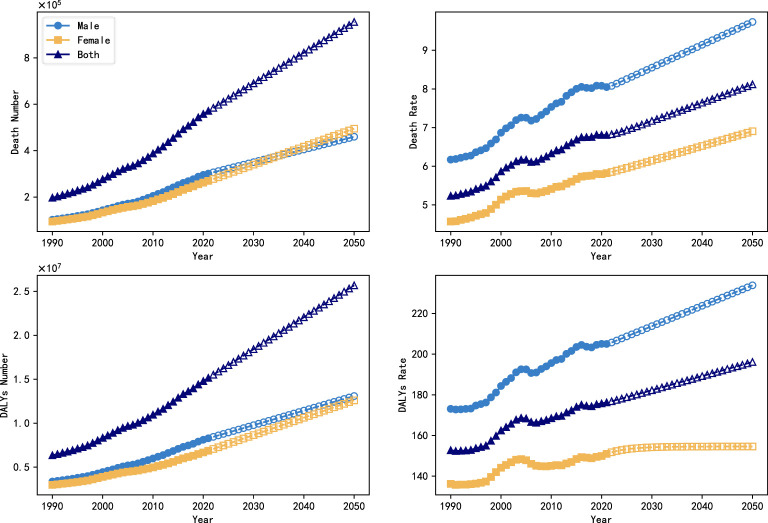
Trends in the global burden of DN (number of deaths, mortality, DALYs, DALYs rate) between 1990 and 2050.

In this paper, an ARIMA model was used to predict trends in the global burden of DN (number of deaths, mortality, DALYs, DALYs rate) from 2022 to 2050, where age-standardized data were used for the mortality and DALYs rate. The results of the model predictions are shown in **Figure 1**. From 1990 to 2021, the global burden of DN increased significantly, with the number of deaths increasing from 197.27 million to 571.29 million, and the number of DALYs increasing from 635.044 million to 151.5 million, a 1.89-fold and 1.38-fold increase, respectively. The global mortality rate increased from 5.233 per 100,000 to 6.807 per 100,000, and the rate of DALYs increased from 152.759 per 100,000 to 176.286 per 100,000.

**Figure 2:**
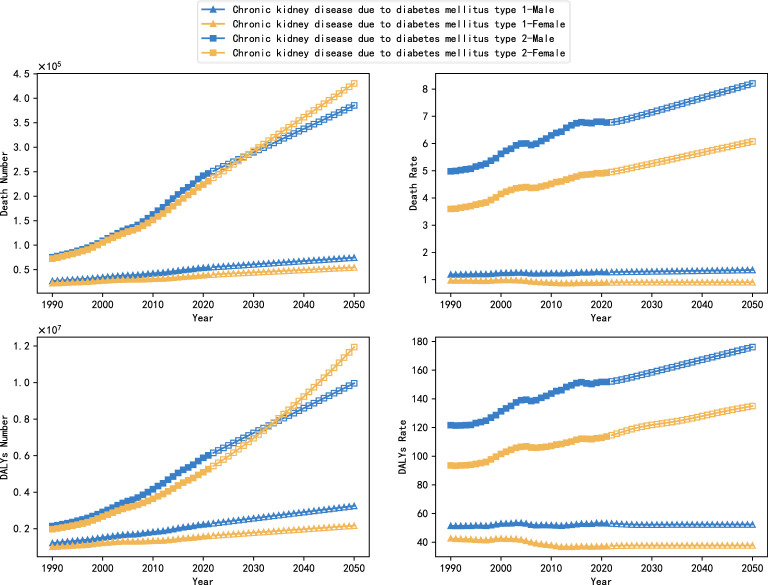
Trends in the global burden of DN by subtype and gender, 1990-2050.

**Figure 2** shows the global burden of DN by sex and subtype between 1990 and 2050, with projections from the ARIMA model for 2022 to 2050. The number of deaths attributable to type 2 DN was 75,450,000 and 72,520,000 deaths in 1990, compared with 26,920,000 deaths from type 1 DN and 22,380,000, and this gap widens further by 2021, with deaths from type 2 DN rising to 246,600,000 and 230,670,000 deaths, about three times as many as in 1990, while type 1 DN is increasing slowly, to only 54,600,000 and 39,420,000 deaths. In terms of gender differences, the mortality and DALYs rates of women were lower than those of men, especially in type 2 DN, which was 4.926/100,000 and 113.671/100,000 in 2021, compared with 6.770/100,000 and 151.813/100,000 in men. However, no significant gender differences were shown in the trend of change.

The ARIMA model projections indicate that the overall burden of type 1 DN will stabilize from 2022 to 2050, with a slow increase in the number of deaths and the value of DALYs, while the death rate and the rate of DALYs will remain more or less unchanged. However, the burden of type 2 DN will continue to increase, and in particular, the number of deaths and values of DALYs in females will exceed those in male patients, with projected values of 430,311,000 and 11,942,950,000 in 2050, compared with projected values of only 385,450,000 and 9,962,080,000 in males.

**Figure 3:**
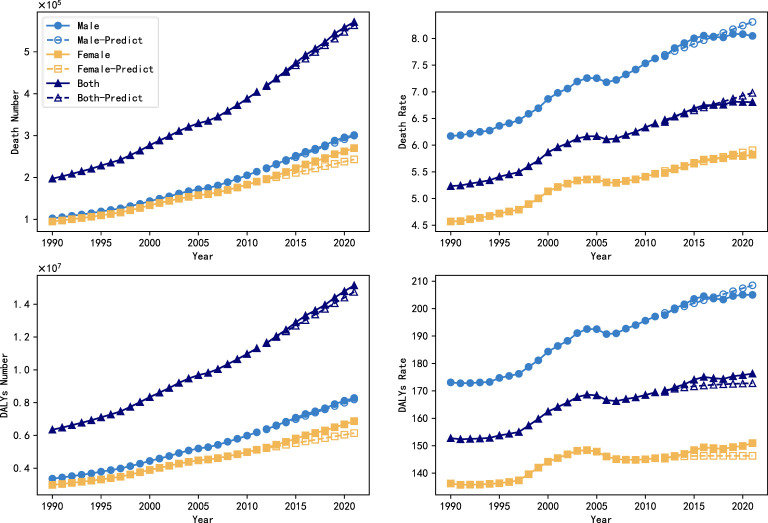
Backtesting of projections of the global burden of DN from 1990 to 2021.

In order to assess the accuracy of ARIMA model in predicting the trend of global diabetic nephropathy burden, this study further used the data from 1990 to 2011 to predict the trend of diabetic nephropathy burden change from 2012 to 2021 using ARIMA model, which was analyzed in comparison with the real data. **Figure 3** shows the predicted results and the true trend of change in the global burden of DN from 1990 to 2021, where the solid curves indicate the true values and the hollow curves indicate the predicted values from the ARIMA model. Subsequently, the predictive effectiveness of the model was systematically analyzed in this study using three commonly used assessment metrics, namely root mean square error (RMSE), mean absolute percentage error (MAPE) and coefficient of determination (R²), and the results are shown in **Table 3**. The results showed that the model had high prediction accuracy, in which the RMSE value was low, the MAPE value was controlled within a reasonable range, and the R² value was close to 1. These results fully demonstrated the applicability and stability of the ARIMA model in the task of diabetic nephropathy prediction, and at the same time provided data support and theoretical basis for the future use of the model for the prediction of long-term burden trends.

**Table 3:** Results of the ARIMA model for predicting and assessing the burden of DN metrics.

	RMSE	MAPE	R^2^
Death Number	7.371×103	1.265	0.993
Death Rate	0.074	0.827	0.992
DALYs Number	2.602×105	1.629	0.992
DALYs Rate	2.366	1.220	0.989

### 3.4 ARIMAX model based on factors of medical technology advancement

The ARIMA model makes predictions based only on past data and does not consider any external factors, so its predictions reflect only the basic trends in the burden of diabetic kidney disease and fail to capture the potential impact of advances in medical technology. The burden of DN is influenced by a variety of factors, including medical technology advances and health policy changes, which are among the factors that improve the burden of disease and the health status of patients. This study further considered the potential impact of medical technology advances on the burden of diabetic nephropathy. In order to incorporate this into a predictive model to simulate a scenario where the burden of DN may gradually ease with advances in medical technology, treatments, and devices, this study used the ARIMAX model.

This study presents a total of two hypothesized results. For the description of the factor of technological progress, this study first assumes that technological progress gradually increases year by year and adopts the S-type (Logistic) growth model to reflect this trend, i.e., it grows slowly at the initial stage, and then accelerates and then tends to saturate as the technology gradually matures and is widely used. Under the assumption that the progress in the level of technology conforms to an S-shaped curve, the expected growth rates of the indicators of the burden of diabetic nephropathy all show a slowly decreasing trend with the gradual promotion of technological progress, especially in the later stages, when there is basically no longer an increase in the number of deaths and the number of DALYs, and the number of deaths of females shows a more pronounced downward trend. In order to assess the impact of technological progress, in comparison with the ARIMA model, the predicted values of the ARIMAX model after taking into account technological progress are 634,630,000 and 7,745/100,000 people for the number of deaths and mortality rates, and 18,194,370,000 and 1,905,011/100,000 people for the number of DALYs and the rate of DALYs, with a significant reduction in the burden in 2050, It illustrates the importance of further spreading the technology of disease prevention and treatment.

This study also uses a proxy variable related to technological advances, namely, global national health expenditure data (https://vizhub.healthdata.org/fgh/) from 1995 to 2021, as an indirect indicator. Global national health expenditure data include development assistance funds, domestic health expenditures, and other expenditures for disease treatment and prevention, health policy development, and other expenditures, which can indirectly characterize global medical technology investment and policy development. When using the global health expenditure data as a proxy for technology level, a comparison of the ARIMAX and ARIMA models shows that they are highly consistent in predicting the trend of disease burden, and both show that the global burden of diabetic nephropathy will continue to show an increasing trend in the future. This may be because the increasing trend in the burden of diabetic kidney disease may be driven by multiple factors, such as population aging and the increasing incidence of diabetes, which are not yet sufficiently counteracted by technological advances in the short term.

**Figure 4:**
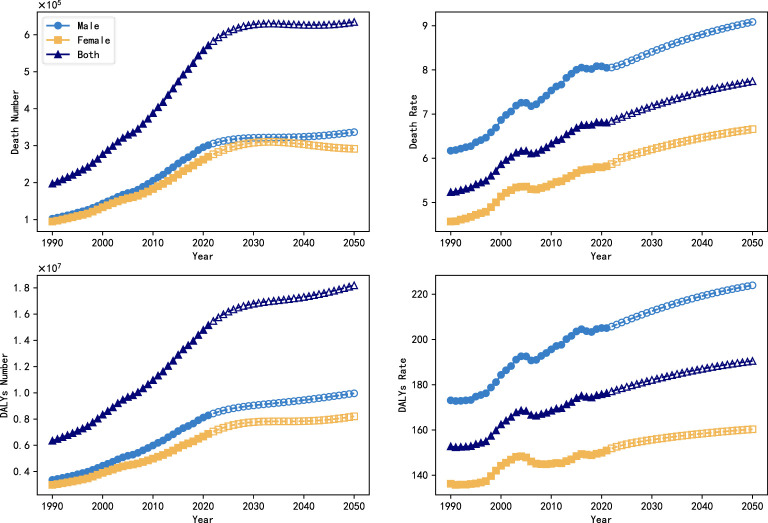
Trends in the global burden of DN with the introduction of a technological progress factor (S-growth), 1990-2050.

**Figure 5:**
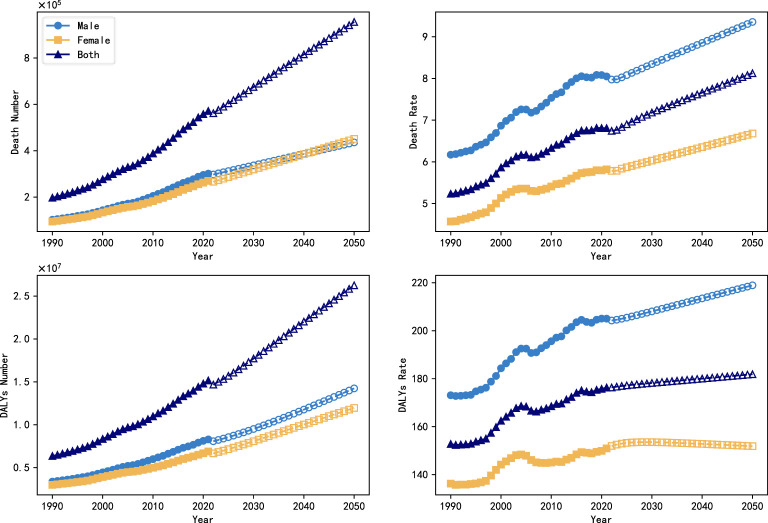
Trends in the global burden of DN with the introduction of technological advances (proxy variables), 1990-2050.

The results of S-growth model-based projections of the burden of DN for the period from 2022 to 2050 are shown in **Figure 4**, and the results of the proxy-variable-based projections are shown in **Figure 5**.

### 3.5 Burden of diabetic nephropathy in different SDI countries in 2021

**Figure 6:**
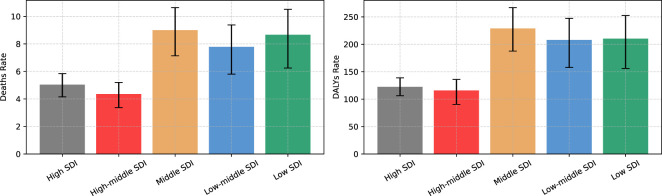
Burden of DN (mortality and DALYs rates) in different SDI countries in 2021.

**Figure 6** analyzes the DN mortality and DALYs rates for different SDI countries in 2021. The results showed that the highest mortality and DALYs rates in 2021 were in the medium SDI subgroup and the lowest were in the medium-high SDI subgroup, with mortality rates of 8.999/100,000 and 4.359/100,000, and DALYs rates of 228.950/100,000 and 115.956/100,000, respectively. In high SDI countries, the mortality and DALYs rates for DN were 5.040/100,000 and 122.472/1,900,000, while in low SDI countries, the corresponding values were as high as 8.668/100,000 and 210.354/100,000.

### 3.6 Changes in the burden of DN in different SDI countries

**Figure 7:**
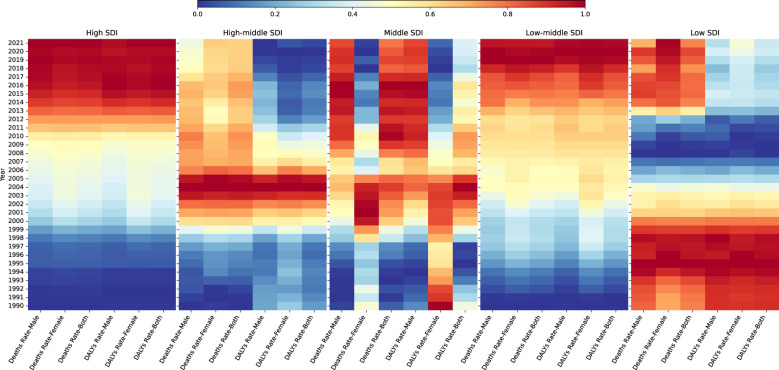
Changes in the burden of DN in different SDI countries from 1990 to 2021.

**Figure 7** shows trends in DN mortality and DALYs rates in different SDI countries from 1990 to 2021, divided by different genders. Where each block represents an SDI subgroup, Each row represents the year, each column represents the different burden indicator and gender combination, and the color of each square represents the value of that indicator normalized within that combination, with blue indicating low burden and red indicating high burden. Overall, countries in different SDI subgroups show different relative trends in the burden of DN, with some gender differences. High SDI countries and low to medium SDI countries showed a consistent pattern in the burden of DN, with consistently increasing rates of mortality and DALYs across genders. The burden in high and medium SDI countries was relatively low until 2000, increased sharply to a maximum between 2000 and 2010, and then declined gradually from 2010 onwards, with DALYs rates declining significantly more than mortality rates, and DALYs rates in 2021 having declined to a level less than that in 1990. Low SDI countries show a different trend, with both mortality and DALYs rates showing a higher burden before 2000, a significant reduction in burden between then and 2012, and a slight rebound after 2012, with the burden of mortality increasing significantly more than DALYs rates. Trends in burden in the medium SDI countries present a more complex picture, with clear gender differences, with male mortality showing a gradual increase and female mortality showing the greatest burden from 2000 to 2005, with an increasing trend for males and an opposite, decreasing trend for females in the DALYs rate.

### 3.7 EAPCs for the burden of DN

**Figure 8:**
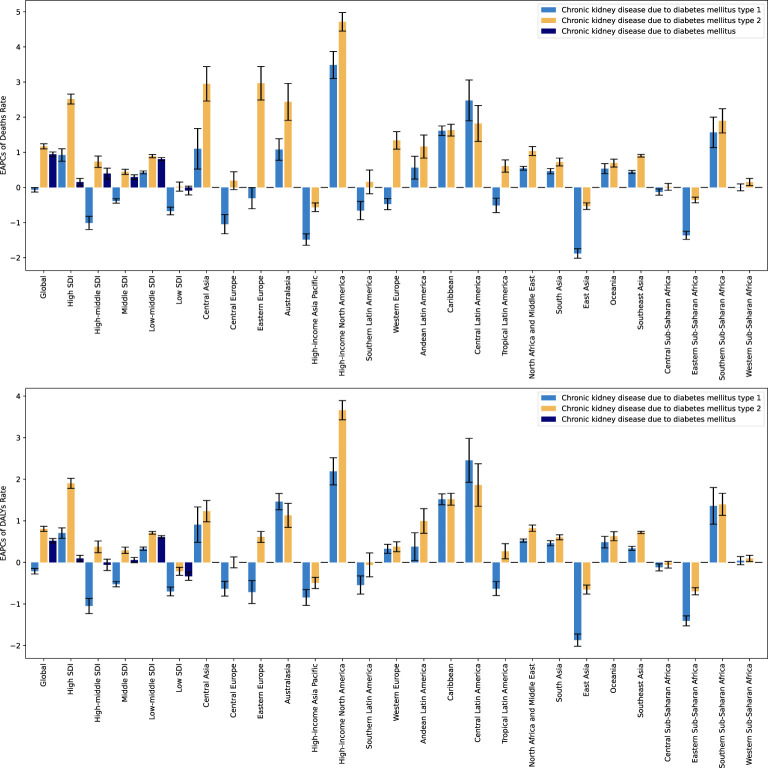
EAPCs for the burden of DN and its subtypes, 1990 to 2021.

**Figure 8** analyzes EAPCs of mortality and DALYs rates for DN and its subtypes in different regions of the world from 1990 to 2021, including global trends, different SDI countries, and 21 different regions. The results show that mortality and DALYs rates for type 2 DN have continued to increase consistently and rapidly over the past decades, while type 1 DN has slowly declined. Specifically, type 2 DN had the highest EAPC values and the fastest increases in mortality and DALYs rates in high SDI countries, indicating a significant increase in the burden of DN and a high average annual growth rate in these countries. In contrast, the smallest EAPC values were found in type 1 DN in medium- and high-SDI countries, with the fastest decreases, which may be related to the successful implementation of more effective diabetes prevention and control strategies in these countries. By region, there is an overall increasing trend in mortality and rates of DALYs, especially for type 2 DN, worldwide and in most SDI regions. High-income North America has the highest EAPC values and the fastest-growing burden of DN, while Central Asia, Eastern Europe, and Australasia also show faster growth in the burden of type 2 DN. Countries or regions experiencing faster growth in type 1 diabetes nephropathy include High-income North America, Central Latin America, and Southern Sub-Saharan Africa, while those experiencing faster declines include High-income Asia-Pacific. Countries or regions with faster declines include High-income Asia Pacific, East Asia, and Eastern Sub-Saharan Africa. Other countries or regions have smaller EAPC values and remain stable overall.

### 3.8 Global burden of DN and distribution of EAPCs by country and region in 2021

**Figure 9:**
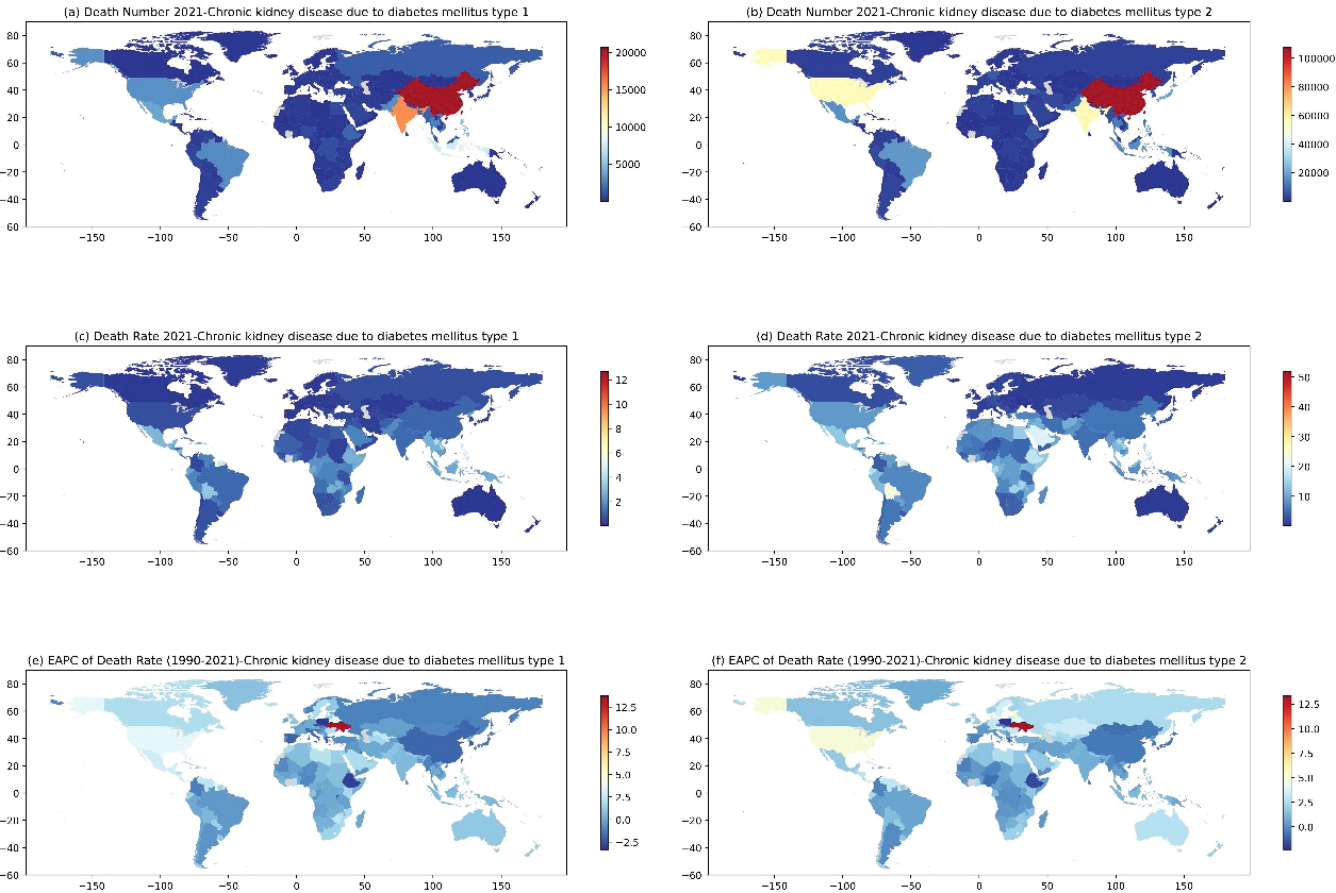
Number of deaths from CKD by T1DM **(A)** and T2DM **(B)**, mortality rates from CKD by T1DM **(C)** and T2DM **(D)** by countries and regions worldwide in 2021, and the distribution of EAPC of death rate corresponding to CKD by T1DM **(E)** and T2DM **(F)** from 1990 to 2021.

**Figure 9** illustrates the distribution of deaths, mortality rates, and EAPCs for DN (type 1 and type 2) across countries and regions globally in 2021. Significant differences in the burden of DN can be found in 204 different countries and regions around the world.

**Figures 9A, B** represent the global heat map of DN deaths, China and India are the two countries with the highest number of deaths, with type 1 DN and type 2 DN deaths amounting to 20,690,000 and 107,650,000 in China, and 15,466,000 and 56,200,000 in India, respectively, and in addition to the US, which had a death toll from type 2 DN of 55.21 million. San Marino, Niue, and Tokelau recorded the fewest deaths. **Figures 9C, D** represent the global heat map of DN mortality, with countries with high mortality rates for type 1 DN including American Samoa, Nauru, Micronesia (Federated States of), and the Marshall Islands, all with mortality rates above 10/100,000 people. Countries with high mortality rates for type 2 DN include American Samoa, the Northern Mariana Islands, and Nauru, all with mortality rates above 40/100,000 people. The countries with the lowest mortality rates were Slovenia (0.056/100,000) and Ukraine (0.189/100,000). In terms of EAPCs, the most significant increase in type 1 DN mortality was in Ukraine with an EAPC value of 13.776, and the most significant decrease was in Poland with an EAPC value of -3.341, and the most significant increase in type 2 DN mortality was also in Ukraine with an EAPC value of 13.373, and the most significant decrease was in Cyprus. The most significant increase in type 2 DN mortality was also in Ukraine with an EAPC value of 13.373, and the most significant decrease was in Cyprus with an EAPC value of -2.320, while Armenia and Latvia also showed high EAPC values of 7.805 and 5.605, respectively.

### 3.9 Global burden of DN by age

**Figure 10:**
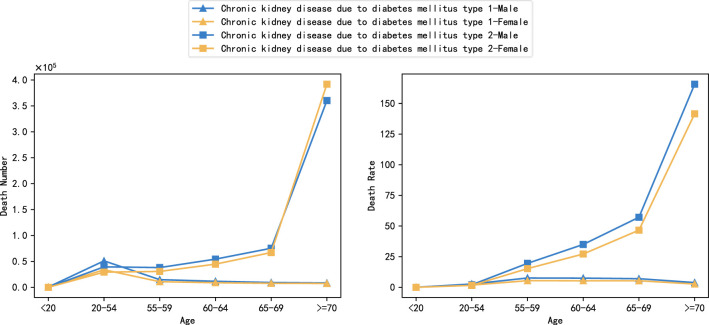
Global burden of DN by age, 2021 (number of deaths and mortality).

**Figure 10** shows the global distribution of deaths and mortality from DN in 2021 by age, considering six age groups:<20 years, 20-54 years, 55-59 years, 60-64 years, 65-69 years, and >=70 years, and comparing type 1 and type 2 DN by gender, respectively. The data showed that although the number of deaths from type 1 DN varied somewhat between age groups, the overall correlation with age was not strong, with the highest number of deaths occurring in the 20-54-year age group, with 51,002,000 males and 33,810,000 females. Mortality was mainly distributed between the ages of 55-69 years, with no significant correlation with changes in age groups, and small fluctuations between age groups. The mortality rate rises with age from 55 years onwards, showing a clear age dependence, The highest mortality rate was found over 70 years old, reaching 1,657,340/100,000 and 1,415,380/100,000 for males and females, respectively. The study showed that there was no significant difference in the correlation between the death burden and age between men and women, and the trend was consistent for both males and females, with the extent of the burden being consistently slightly higher for males than for females within the same age group, except the number of deaths from type 2 DN at the age of 70 years or older.

### 3.10 Burden of DN due to metabolic factors in different age groups

**Figure 11:**
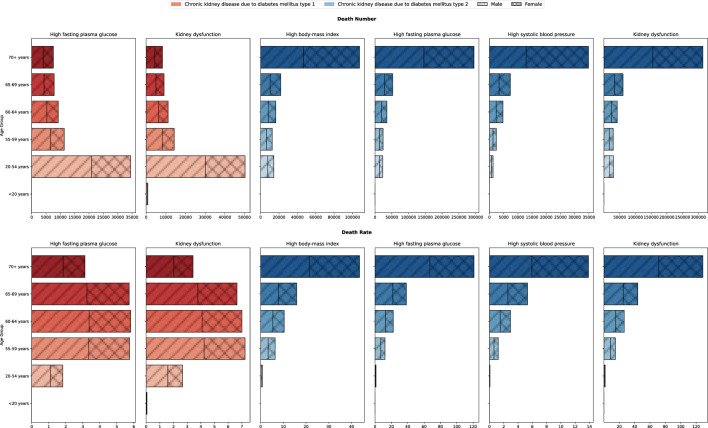
Burden of DN due to metabolic factors in different age groups, 2021.

**Figure 11** shows the global burden of type 1 and type 2 DN due to metabolic factors in different age groups in 2021, covering comparisons of the number of deaths and mortality rates for four metabolic factors: high body-mass index, high fasting plasma glucose, high systolic blood pressure, and kidney dysfunction. The results showed that the burden of DN differed significantly between age groups, but the effect of metabolic factors on the burden was more similar across age groups and did not show significant variability. The trend of distribution of burden due to different metabolic factors was consistent across age groups, suggesting that the specific impact of metabolic factors was relatively balanced across age groups. For type 1 DN, high fasting glucose, and renal dysfunction were the main causative factors, especially in the age group of 20-54 years, the contribution of metabolic factors to the number of deaths was significantly higher than that of other age groups, reaching 34,520,000 and 50,280,000, respectively. However, the distribution of mortality from type 1 DN in all age groups differed from the number of deaths, favoring a more homogeneous distribution, mainly concentrated in the ages 55-59, 60-64, and 65-69. The differences in the distribution of deaths and mortality may reflect the physiological status of patients in different age groups and the different mechanisms of disease progression. For type 2 DN, Kidney dysfunction was the factor causing the highest mortality rate in type 2 DN, with 121.245/100,000 and 94.886/100,000 for men and women, respectively, and the factor with the lowest mortality rate was high systolic blood pressure, with 10.680/100,000 for men and women, respectively. The lowest mortality factor was high systolic blood pressure (10.680/100,000 and 12.784/100,000 for men and women, respectively).

## 4 Discussion

In this study, we find that between 1990 and 2021, the global CKD due to Diabetes Mellitus deaths, mortality, and DALYS rates increased significantly. Type 2 DN demonstrated a major contribution to the overall burden of disease, with significantly higher values than type 1 DN for the four burden indicators, with an expanding trend from year to year. Type 2 DN is increasing at a much faster rate than type 1 DN and the burden of disease is more pronounced in men. With global aging and population growth, DALY for CKD has been increasing year by year, coinciding with the findings of this study (21). Globally, the prevalence of type 2 diabetes accounts for more than 90% of all cases of diabetes, and type 2 diabetes is also the leading cause of DN (22). This data indirectly explains why the rise in type 2 DN is more pronounced.

This study further used the ARIMA prediction model to estimate disease trends. ARIMA model projections indicate that in the future, without effective public health interventions, the global burden of DN will continue to increase significantly, with the number of deaths projected by 2050 to reach 954,800,000, the number of DALYs will increase to 25,691,300, the mortality rate will reach 8.118/100,000 people, and the DALYs rate will reach 196.143/100,000 people. There is a clear gender difference in this increasing trend, where the data indicate that the mortality rate and DALYs rate are significantly higher in males than in females, and the ARIMA model predictions show that the DALYs rate in males is increasing significantly faster than that in females. Since “male” and “smoking history” are two factors that contribute to increased cardiovascular risk in CKD patients, they further increase the risk of disability and death (23). However, according to the ARIMA model, CKD caused by type 2 in women will have more deaths and DALYs than men in the future. Therefore, there is also a need for increased attention to type 2 DN disease in the female population.

The rates of DN mortality and DALYs were significantly lower in high SDI and medium-high SDI countries than in the other three subgroups (medium SDI, medium-low SDI, and low SDI). The significant differences in trends in the burden of DN in different SDI countries illustrate the differences in vulnerability and coping capacity in dealing with the burden of diabetes in countries at different levels of socioeconomic development. Previous studies, together with the present study, have emphasized the important impact of the level of socioeconomic development on the burden of diabetes and its associated complications, with a greater increase in the number of people with diabetes in low- and middle-income countries (24). In the future, more targeted interventions should be developed for these regions to enhance the prevention and management of diabetes in order to effectively control the burden of DN. For example, knowledge of the prevention of diabetes and diabetic nephropathy has been popularized through community activities, online courses, and media campaigns (25). Promotion of a healthy lifestyle, weight control, moderate exercise, smoking cessation, Mediterranean diet and restriction of sodium intake are also required, as well as appropriate lipid-lowering therapy and medication to control blood pressure (26). Promote the use of lower-cost testing techniques, such as urine microalbumin strip testing, in resource-limited areas (27). However, in patients with type 2 diabetes mellitus, decreased renal function suggests the risk of non-albuminuric diabetic kidney disease, even if the urinary albumin level is normal. Therefore, we need to pay attention to both the estimated glomerular filtration rate and urine albumin index (28).

China and India are the two countries with the highest number of deaths, which may be related to their larger populations. A Chinese study showed that the prevalence of CKD is high but awareness of disease prevention is low, and that developing countries are experiencing rapid economic development but a marked lack of health awareness (29). There are significant differences in the level of medical care in different regions of the developing world, and telemedicine technology can be utilized to provide medical care in remote areas and reduce geographical barriers to patient access. Predictive modeling is also recommended to estimate the risk of undiagnosed CKD and the risk of CKD progression in diabetic patients. At the same time, a multidisciplinary teamwork model, including endocrinology, nephrology, and cardiovascular medicine, was promoted to provide comprehensive management services (27).

The burden of death from the disease is significantly related to age, with a sharp increase in deaths especially in the older age group of 70 years and older, and fewer deaths in the younger age group. Previous literature has reported that diabetes mellitus and CKD are the leading causes of DALY in the elderly population, and the progressive decline in body functions with age increases morbidity and mortality from DN (30).

In this study, besides analyzing the burden of disease, we also analyzed the effect of different metabolic factors on the rate of disease mortality, DALYs. The distribution of the burden of different metabolic factors among age groups was relatively consistent and did not show significant differences. The effects of the four metabolic factors on the number of deaths and mortality rates were positively correlated with age, with the burden of disease being heaviest in the age group over 70 years, decreasing with age, and being very low in the population under 20 years of age. This phenomenon suggests the key role of metabolic disorders in the progression of nephropathy in older diabetic patients. In addition, although there were slight differences in burden manifestations between males and females at different ages, the overall trends were similar, suggesting that gender does not constitute a major influence in metabolic factor-induced mortality and deaths from DN. For type 1 DN, high fasting glucose, and renal dysfunction were the main causative factors. Hyperglycemia can lead to the accumulation of glycosylation end products (AGEs), which activate oxidative stress and inflammatory responses in renal tissues, thereby triggering glomerular hyperfiltration and tubulointerstitial injury (31). Inflammatory factors such as interleukin-6 (IL-6) and tumor necrosis factor-α (TNF-α) can activate glomerular mesangial cells, further aggravating renal impairment (32). Therefore, controlling blood glucose levels is essential to slow down the progression of type 1 diabetic nephropathy. For type 2 DN, Kidney dysfunction was the factor causing the highest mortality rate in type 2 DN. The pathogenesis of T2DN is not only related to hyperglycemia but also closely related to insulin resistance and lipid metabolism disorders. These metabolic abnormalities can lead to glomerular hyperfiltration and tubulointerstitial fibrosis. Inflammation also plays a key role in the progression of T2DN, and the increased expression of inflammatory factors such as monocyte chemotactic protein-1 (MCP-1) and kidney injury molecule-1 (KIM-1) in renal tissues is closely related to the deterioration of renal function (33). In addition, localized renin-angiotensin system (RAS) activation in the kidney is one of the important pathophysiological mechanisms of T2DN, leading to glomerular hypertension and tubular injury.

## 5 Conclusions

The current study found that the global burden of DN increased progressively from 1990 to 2021, and in the absence of interventions, the global burden of DN is projected to continue to rise annually from 2022 to 2050, placing even greater stress on the global health system in the future, especially in men with DN. The burden of DN is consistently higher in intermediate SDI countries. Therefore, more accurate and cost-effective diagnostic tools and interventions for the effective management of diabetes and its complications are needed in the future, especially in low- and middle-income countries with poor healthcare resources.

## Data Availability

Publicly available datasets were analyzed in this study. This data can be found here: http://ghdx.healthdata.org/gbd-results-tool.
